# Clinical risk factors and outcomes of carbapenem-resistant *Escherichia coli* nosocomial infections in a Chinese teaching hospital: a retrospective study from 2013 to 2020

**DOI:** 10.1128/spectrum.04228-23

**Published:** 2024-05-30

**Authors:** Haifang Kong, Zhidong Hu, Longtao Zhang, Qianqian Chen, Ling Yang, Jin Li, Bin Tian, Yamin Chai, Xuequan Feng

**Affiliations:** 1 Department of Laboratory Medicine, Tianjin Medical University General Hospital, Tianjin, China; 2 Tianjin Medical University General Hospital, Tianjin, China; 3 Tianjin First Central Hospital of Nankai University, Tianjin, China; City of Hope, Duarte, California, USA

**Keywords:** carbapenem-resistant* Escherichia coli*, risk factors, nosocomial infection, clinical outcomes

## Abstract

The emergence of carbapenem-resistant *Escherichia coli* strains poses a considerable challenge to global public health, and little is known about carbapenemase-producing *E. coli* strains in Tianjin, China. This study aimed to investigate the risk factors for infections with carbapenem-resistant *E. coli* (CREC) strains. This retrospective case–control study was conducted at a tertiary teaching hospital. A total of 134 CREC clinical isolates were collected from the General Hospital of Tianjin Medical University between 2013 and 2020. The control group was selected at a ratio of 1:1 from patients with nosocomial carbapenem-susceptible *E. coli* infection. Risk factors for nosocomial CREC infection and clinical outcomes were analyzed using univariate and multivariate analyses. Multivariate analysis revealed that cephalosporin exposure (odd ratio OR = 2.01), carbapenem exposure (OR = 1.96), glucocorticoid exposure (OR = 32.45), and surgical history (OR = 3.26) were independent risk factors for CREC infection. The in-hospital mortality rate in the CREC group was 29.1%, and age >65 years (OR = 3.19), carbapenem exposure (OR = 3.54), and central venous catheter insertion (OR = 4.19) were independent risk factors for in-hospital mortality in patients with CREC infections. Several factors were identified in the development of nosocomial CREC infections. The CREC isolates were resistant to most antibiotics. Reducing CREC mortality requires a comprehensive consideration of appropriate antibiotic use, underlying diseases, and invasive procedures.

**IMPORTANCE:**

*Escherichia coli* is an opportunistic pathogen that causes severe hospital-acquired infections. The spread of carbapenem-resistant *E. coli* is a global threat to public health, and only a few antibiotics are effective against these infections. Consequently, these infections are usually associated with poor prognosis and high mortality. Therefore, understanding the risk factors associated with the causes and outcomes of these infections is crucial to reduce their incidence and initiate appropriate therapies. In our study, several factors were found to be involved in nosocomial carbapenem-resistant *E. coli* (CREC) infections, and CREC isolates were resistant to most antibiotics. Reducing CREC mortality needs a comprehensive consideration of whether antibiotics are used appropriately, underlying diseases, and invasive interventions. These findings provide valuable evidence for the development of anti-infective therapy, infection prevention, and control of CREC-positive infections.

## INTRODUCTION

*Enterobacterales* are the most common pathogenic Gram-negative bacteria responsible for severe nosocomial infections (1, 2). In particular, the worldwide occurrence of carbapenem-resistant *Enterobacterales* (CRE) infections has increased in recent years, including China, representing a considerable public health challenge (3–6). In early 2017, the World Health Organization (WHO) released a list of drug-resistant bacteria, including CRE, that pose a substantial threat to human health, highlighting the urgent need to develop new antibiotics (7).

Carbapenem has long been used as a potent agent against Gram-negative bacilli. However, few treatment options are available for CRE infections, which can result in high mortality (8). In recent years, carbapenem-resistant *Escherichia coli* (CREC), a class of CRE, has become a major threat in hospitals worldwide. Carbapenem resistance in *Escherichia coli* is mainly caused by plasmid-encoded carbapenemases, which easily spread among bacteria, resulting in outbreaks of nosocomial CREC infections (9–11). Only a few antibiotics, such as tigecycline and polymyxins, are effective against CREC. Although new antibiotics, such as ceftazidime–avibactam, have been used for the treatment of CRE infections, they remain ineffective against New Delhi metallo-beta-lactamase (NDM)-producing CREC (12, 13).

A global survey of CREC isolates was conducted for characterizing the carbapenemase types (14). *Klebsiella pneumoniae* carbapenemase (KPC) and NDM have emerged as the most prevalent carbapenemases worldwide, with frequencies of 55/343 and 53/343, respectively. Notably, KPC was dominant in the United States, accounting for 17% of isolates (15). Conversely, OXA-48-like producers constituted the majority in Europe, representing 56% of the isolates, followed by NDM (26%) and KPC (18%) (16). The China Antimicrobial Surveillance Network (CHINET) revealed that NDM-5 is the primary carbapenemase in CREC (74.5%), with 49.0% of the CRE strains isolated from pediatric patients exhibiting NDM production (17). Based on multiple characteristics, the global CREC population is found to be highly diverse and varies significantly by the geographical region. This poses challenges for prevention and management and necessitates ongoing surveillance. Thus, an understanding of the risk factors associated with the causes and outcomes of these infections is required to reduce their incidence and initiate appropriate therapies.

Several previous studies have investigated the risk factors for CRE infections (18–21); however, few have specifically evaluated the risk factors for CREC acquisition. Therefore, we performed a case–control study to evaluate the risk factors of CREC infection among inpatients at a teaching hospital in China.

## MATERIALS AND METHODS

### Patient population and study design

**Fig 1:**
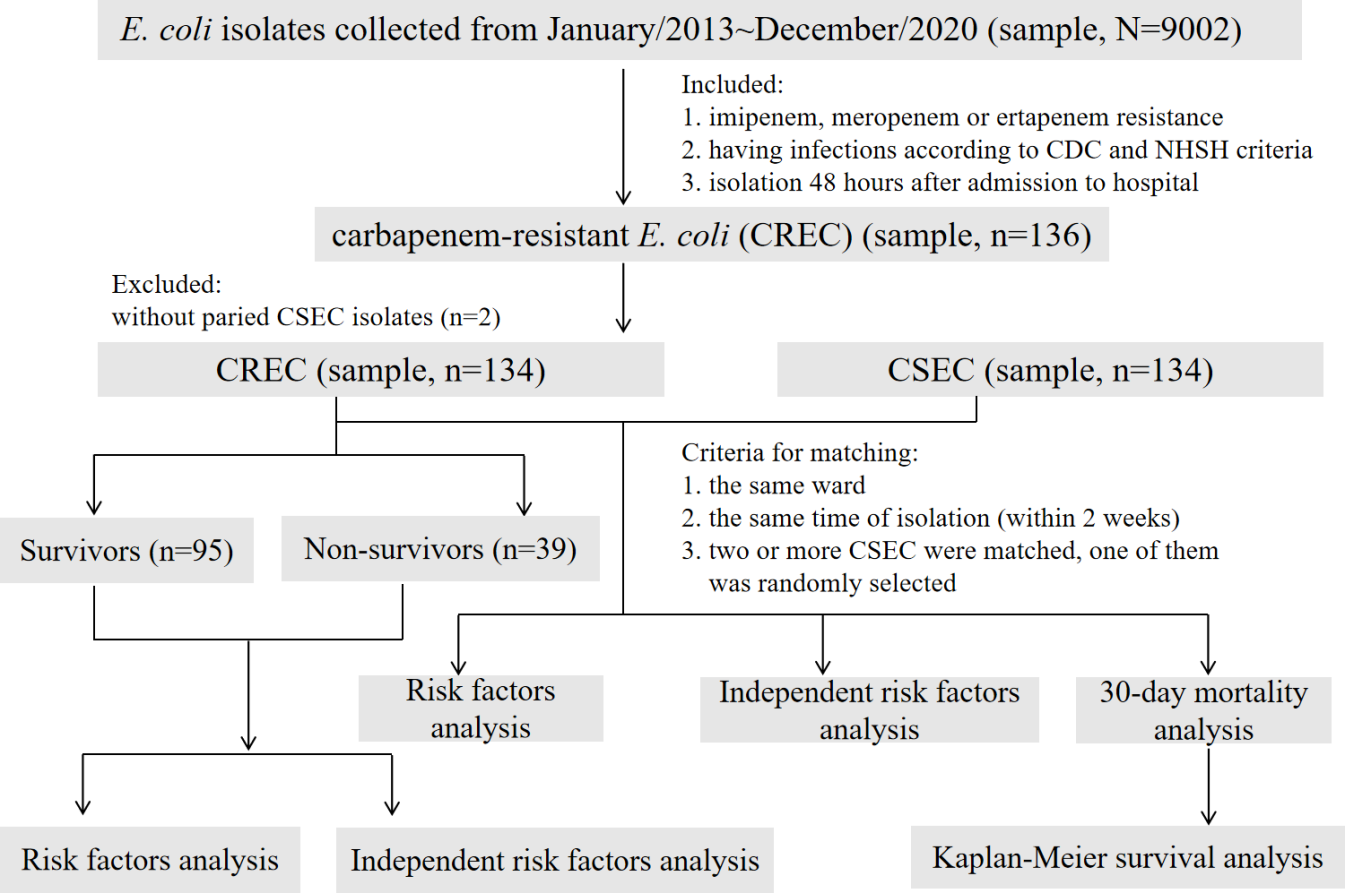
Flowchart of sample selection, comparison, and value. From 9,002 samples, a total of 134 CREC isolates were selected for further analysis. A matched CSEC control group was used to analyze the risk factors for CREC infection, and risk factors for 30-day mortality were also analyzed. According to the final outcome of hospitalized patients, the 134 CREC isolates were divided into survivors and non-survivors for analyzing the risk factors for in-hospital mortality. Abbreviations: CREC, carbapenem -resistant *Escherichia coli*; CSEC, carbapenem-sensitive *Escherichia coli*; CDC, Centers for Disease Control and Prevention; NHSN, National Healthcare Safety Network.

This was a single-center, 8-year, continuous study of CREC strains, using specific inclusion/ exclusion criteria (Fig. 1). A total of 134 clinical CREC isolates were collected from Tianjin Medical University General Hospital, a 2,468-bed tertiary-care hospital in China, from January 2013 to December 2020. Only the first isolates of CREC or carbapenem-sensitive *E. coli* (CSEC) collected from multiple sites or on multiple dates were included in the analysis. Nosocomial CREC or CSEC infections were defined as isolates obtained 48 hours after hospital admission.

### Case criteria

A case–control study was conducted to assess the risk factors and clinical outcomes of CREC infection (22). For each patient with a CREC infection, we selected a control patient from a pool of patients with CSEC infections.

CREC isolates resistant to any carbapenem (imipenem, meropenem, and ertapenem) were considered resistant. Carbapenem (imipenem and/or meropenem) resistance was confirmed using the Kirby–Bauer (K–B) method according to the manufacturer’s instructions (Thermo Fisher Scientific, United Kingdom).

The CSEC group was selected from the same ward as the source population during the same period as the CREC group (within 2 weeks); if two or more controls were matched, one was randomly selected.

### Micro-biologic methods

All isolates were identified using matrix-assisted laser desorption ionization time-of-flight mass spectrometry (bioMerieux, France). An automated broth microdilution method (VItek2; bioMerieux) was used for susceptibility testing. The Clinical and Laboratory Standards Institute (CLSI-M100) document was used to interpret the antimicrobial susceptibility testing (23). *E. coli* ATCC25922 and *Pseudomonas aeruginosa* ATCC27853 were used as control strains for antimicrobial susceptibility testing.

### Data collection

We reviewed the medical records and collected patient information (20–22). The epidemiology and clinical data of patients were collected, including the department, sex, age, age >65 years, underlying diseases (respiratory system, liver, urinary system, circulatory system, and central nervous diseases; sepsis; digestive system diseases; diabetes mellitus; malignancy; and immunosuppression), antibiotics (third-generation cephalosporin, β-lactam inhibitor compounds, carbapenem, quinolones, tigecycline, and macrolides); glucocorticoids, within 3 months prior to CREC infections; surgical history and invasive procedures (mechanical ventilation, central venous catheter, arterial and urinary catheters, drainage, and gastric tubes) within 1 month prior to a positive culture; and mortality, related to hospitalization (hospital stays prior to CREC isolation, intensive care unit (ICU) stays within 3 months prior to a positive culture, and total duration of hospital stays).

Patients with a positive culture from blood or any other sterile source were defined as having an infection, and patients with positive cultures from respiratory, urine, and surgical wounds were defined as having an infection based on the Centers for Disease Control and Prevention (CDC) and National Healthcare Safety Network (NHSN) criteria (24).

The clinical outcomes were defined as follows: 30-day mortality (within 30 days of the first positive culture), in-hospital mortality (death during hospitalization after the first culture), and total duration of hospital stay (duration from admission to hospital-to-hospital discharge).

### Statistical analysis

Normally distributed continuous variables are presented as mean ± standard deviation (SD) and were compared using a *t*-test. Non-normally distributed continuous variables are presented as medians with interquartile ranges (IQRs) and were compared using the Mann–Whitney U-test. Categorical variables are presented as counts and percentages and were compared using the χ test or Fisher’s exact test.

Univariate analyses were performed for each variable, and those with a *P* < 0.05 were included in a multivariate logistic regression analysis. Statistical significance was set at *P* < 0.05, and SPSS 26.0 was used for statistical analyses.

## RESULTS

### The number of CREC infections

**Fig 2:**
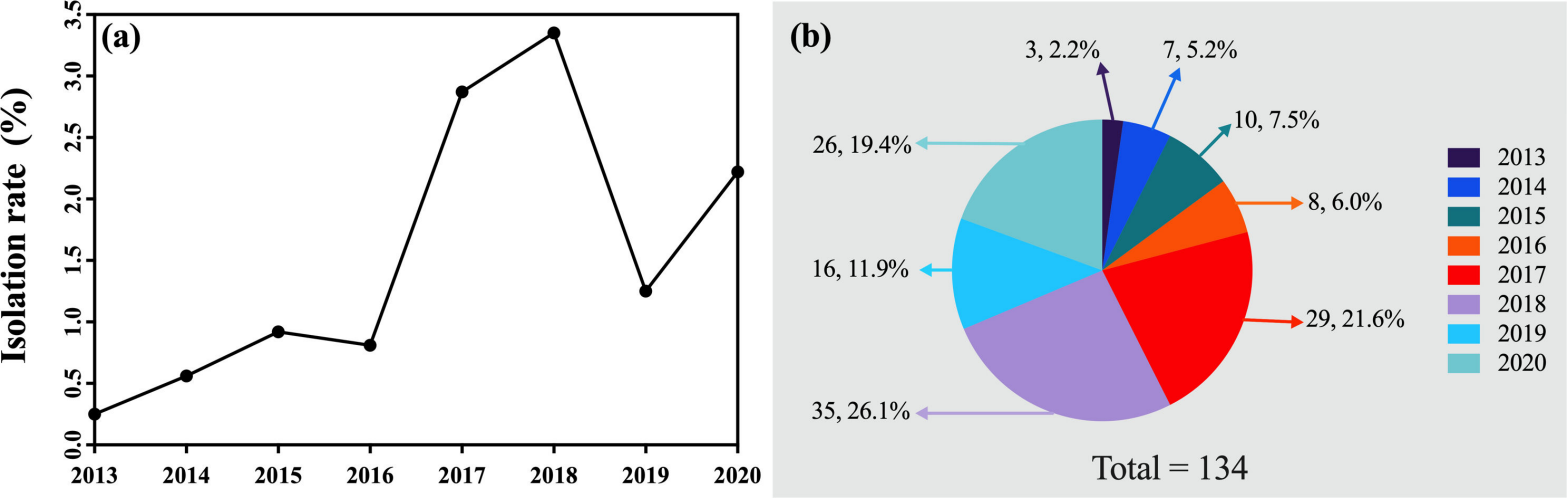
Distribution of carbapenem-resistant E. coli (CREC) strains over the 8-year study period. (a) The isolation rate of CREC from 2012 to 2020 shows the change in isolation rates over the 8-year study period; (b) the number and proportion of CREC strains isolated each year, with the majority of strains being isolated in 2017, 2018, and 2020.

A total of 134 patients with CREC infections were identified during the 8-year study period: three patients in 2013 (isolation rate 0.25%, 3/1,179); seven patients in 2014 (isolation rate 0.56%, 7/1,241); ten patients in 2015 (isolation rate 0.92%, 10/1,084); eight patients in 2016 (isolation rate 0.81%, 8/991); 29 patients in 2017 (isolation rate 2.87%, 29/1,011); 35 patients in 2018 (isolation rate of 3.35%, 35/1,044); 16 patients in 2019 (isolation rate 1.25%, 16/1,280); and 26 patients in 2020 (isolation rate of 2.22%, 26/1,172). (Fig. 2).

### Specimen source ward and source site

A total of 134 patients were included in this study. Most patients infected with CREC were admitted to the ICU (47%, 63/134), and then in general medical wards (36.6%, 49/134), and surgical wards (16.4%, 22/134). The most common types of infections were respiratory tract, urinary tract, and bloodstream infections (Table 1).

**TABLE 1:** The infection sites of carbapenem-resistant *E. coli* (CREC) strains^*a*^

Infection sites	Percentage[%/ (n/N)]	Infection sites	Percentage [%/ (n/N)]
Respiratory tract	46.3 (62/134)	Wound secretion	4.5 (6/134)
Urine	26.8 (36/134)	Vaginal secretion	1.5 (2/134)
Blood	9.7 (13/134)	Bile	1.5 (2/134)
Intra-abdominal	7.5 (10/134)	Others	2.2 (3/134)

^*a*^ Others: one isolate was cervical secretion; two isolates were skin swabs.

### Resistance rate to antibiotics

The antibiotic susceptibility patterns of CREC and CSEC isolates are shown in Table 2. All the CREC isolates were resistant to ampicillin, cefazolin, cefuroxime, ceftriaxone, and ciprofloxacin. The resistance rates of CREC to amoxicillin/clavulanic acid, cefoperazone/sulbactam, piperacillin /tazobactam, ceftazidime, cefepime, aztreonam, levofloxacin, and trimethoprim/sulfamethoxazole were higher than 80%, whereas the resistance rate to amikacin was relatively low.

**TABLE 2:** The antibiotic resistance of CREC and CSEC groups^*a*^

Antibiotics	CREC (*n* = 134)	CSEC (*n* = 134)	*P*
Ampicillin	134/134 (100%)	113/134 (84.3%)	<0.001
Amoxicillin/clavulanic acid	129/134 (96.3%)	15/134 (11.2%)	<0.001
Cefoperazone/sulbactam	126/134 (94.0%)	7/134 (5.2%)	<0.001
Piperacillin/tazobactam	132/134 (98.5%)	6/134 (4.5%)	<0.001
Cefazolin	134/134 (100%)	84/134 (62.7%)	<0.001
Cefuroxime	134/134 (100%)	71/134 (53.0%)	<0.001
Ceftazidime	112/134(83.6%)	31/134(23.1%)	<0.001
Ceftriaxone	134/134(100%)	73/134(54.5%)	<0.001
Cefepime	130/134(97.0%)	35/134(26.1%)	<0.001
Aztreonam	121/134(90.3%)	51/134(38.0%)	<0.001
Amikacin	38/134(28.4%)	2/134(1.5%)	<0.001
Gentamicin	91/134(67.9%)	63/134(47.0%)	0.001
Tobramycin	67/134(50.0%)	24/134(18.0%)	<0.001
Ciprofloxacin	134/134(100%)	94/134(70.1%)	<0.001
Levofloxacin	127/134(94.8%)	82/134(61.2%)	<0.001
Trimethoprim/ sulfamethoxazole	115/134(85.8%)	73/134(54.5%)	<0.001

^*a*^ CREC, carbapenem-resistant *Escherichia coli*; CSEC, carbapenem-sensitive *Escherichia coli*.

### Risk factors for CREC infection

The results of the univariate analyses comparing the demographic and clinical characteristics of patients infected with CREC or CSEC are shown in Tables 3 and 4. The variables associated with CREC infections were as follows: liver diseases (OR = 1.77; 95% CI, 1.25–2.49; *P* = 0.001), circulatory system diseases (OR = 1.31; 95% CI, 1.07–1.62; *P* = 0.009), digestive system diseases (OR = 1.46; 95% CI, 1.02–2.08; *P* = 0.04); exposure to antibiotics, including third-generation cephalosporins (OR = 1.46; 95% CI, 1.08–1.95; *P* = 0.01), carbapenem (OR = 1.60; 95% CI, 1.24–2.05; *P* < 0.001), β-lactam inhibitor compounds (OR = 2.47; 95% CI, 1.42–4.28; *P* = 0.001), quinolones (OR = 1.56; 95% CI, 1.00–2.43; *P* = 0.05), and glucocorticoids (OR = 27; 95% CI, 3.72–195.85; *P* < 0.001), within 3 months prior to a positive culture; surgical history (OR = 1.95; 95% CI, 1.43–2.65; *P* ＜ 0.001), and invasive procedures, including central venous catheter insertion (OR = 1.61; 95% CI, 1.16–2.23; *P* = 0.01), drainage tube insertion (OR = 1.65; 95% CI, 1.08–2.52; *P* = 0.02), and gastric tube insertion (OR = 1.33; 95% CI, 1.01–1.74; *P* = 0.04), within 1 month prior to a positive culture; prior hospital stays (*P* ＜ 0.001), ICU stays (*P* = 0.005), and length of hospital stays (LOS) (*P* = 0.01).

**TABLE 3:** Demographic characteristics of the CREC and CSEC groups^*a*^

	CREC (*N* = 134) (n, %)	CSEC (*N* = 134) (n, %)	χ^2^/ t/U	OR	95% CI	*P*
Sex, male	66 (49)	73 (54)	0.73	0.81	(0.50,1.31)	0.39
Age (years), (mean, SD)	59.8 ± 21.4	62.2 ± 22.4	0.86	—	—	0.38
Age >65	65 (48)	74 (55)	1.21	0.88	(0.70,1.11)	0.27
Prior hospital stays median (IQR)	10.5 (4,23)	5 (3,13)	6272	—	—	<0.001
ICU stays, median(IQR)	4.5 (0,30)	0 (0,11.3)	6854	—	—	0.01
LOS(days),median(IQR)	29.5 (15,55)	19 (11.8,39.5)	6908	—	—	0.01

^*a*^ SD, standard deviation; CREC, carbapenem-resistant *Escherichia coli*; CSEC, carbapenem-sensitive *Escherichia coli*; LOS, length of hospital stays; IQR, interquartile range.

**TABLE 4:** Univariate analyses regarding the risk factors for CREC infections^*a*^

Variable	CREC (*N* = 134) (n, %)	CSEC (*N* = 134) (n, %)	χ^2^/ t/U	OR	95% CI	*P*
Underlying disorder						
Respiratory diseases	74 (55)	59 (44)	3.36	1.25	(0.98,1.59)	0.07
Liver diseases	60 (45)	34 (25)	11.08	1.77	(1.25,2.49)	0.001
Urinary system diseases	66 (49)	57 (43)	1.22	1.16	(0.89,1.50)	0.27
Circulatory system diseases	88 (66)	67 (50)	6.75	1.31	(1.07,1.62)	0.01
Central nervous system diseases	60(45)	59 (44)	0.02	1.02	(0.78,1.33)	0.90
Sepsis	21 (16)	24 (18)	0.24	0.88	(0.51,1.49)	0.62
Digestive system diseases	51 (38)	35 (26)	4.38	1.46	(1.02,2.08)	0.04
Diabetes mellitus	37 (28)	46 (34)	1.41	0.80	(0.56,1.15)	0.23
Malignancy	6 (5)	18 (13)	6.59	0.33	(0.14,0.81)	0.01
Antibiotics exposure within 3 months prior to CREC infection						
Third-generation cephalosporin	65(49)	45 (34)	6.44	1.46	(1.08,1.95)	0.01
β-Lactam inhibitor	83 (62)	52 (39)	14.34	1.60	(1.24,2.05)	<0.001
Carbapenem	37 (27)	15 (11)	11.55	2.47	(1.42,4.28)	0.001
Quinolones	39 (29)	25 (19)	4.02	1.56	(1.00,2.43)	0.05
Tigecycline	14 (10)	6 (5)	3.46	2.33	(0.92,5.89)	0.06
Macrolides	5 (4)	1(1)	2.73	5.00	(0.59,42.23)	0.10
Antifungal agents	9 (7)	8(6)	0.06	1.13	(0.45,2.83)	0.80
Glucocorticoids	27 (20)	1(1)	26.96	27	(3.72,195.85)	<0.001
Surgical history and invasive procedures within 1 month prior to CREC infection						
Surgical history	74 (55)	38 (28)	19.88	1.95	(1.43,2.65)	<0.001
Mechanical ventilation	54 (40)	41 (31)	2.61	1.31	(0.94,1.81)	0.11
Central venous catheter insertion	61 (45)	38 (28)	8.47	1.61	(1.16,2.23)	0.01
Arterial catheters	44 (33)	36 (27)	1.14	1.22	(0.84,1.77)	0.29
Urinary catheter insertion	94 (70)	79 (59)	3.67	1.19	(1.00,1.42)	0.06
Drainage tube insertion	43 (32)	26 (19)	5.64	1.65	(1.08,2.52)	0.02
Gastric tube insertion	69 (52)	52 (39)	4.35	1.33	(1.01,1.74)	0.04
Death	39 (29)	20 (15)	7.85	1.95	(1.20,3.16)	0.01

^*a*^ CREC, carbapenem-resistant *Escherichia coli*; CSEC, carbapenem-sensitive *Escherichia coli*.

Multivariate conditional logistic regression analysis indicated that exposure to third-generation cephalosporin (OR = 2.01; 95% CI, 1.13–3.59; *P* = 0.02), carbapenem (OR = 1.96; 95% CI, 1.10–3.50; *P* = 0.02), and glucocorticoids (OR = 32.45; 95% CI, 4.15–253.60; *P* = 0.001), within 3 months prior to a positive culture, as well as surgical history within 1 month prior to a positive culture (OR = 3.26; 95% CI, 1.80–5.88; *P* ＜ 0.001), were identified as independent risk factors for CREC infection (Table 5).

**TABLE 5:** Multivariate analyses regarding the risk factors for CREC infections

Variable	β	SE	Wald (χ^2^)	OR	95% CI	*P*
Third-generation cephalosporin	0.70	0.30	5.58	2.01	1.13–3.59	0.02
Carbapenem	0.67	0.30	5.19	1.96	1.10–3.50	0.02
Surgical history	1.18	030	15.31	3.26	1.80–5.88	＜0.01
Glucocorticoids	3.48	1.05	11.00	32.45	4.15–253.60	0.001

### Clinical outcomes

**Fig 3:**
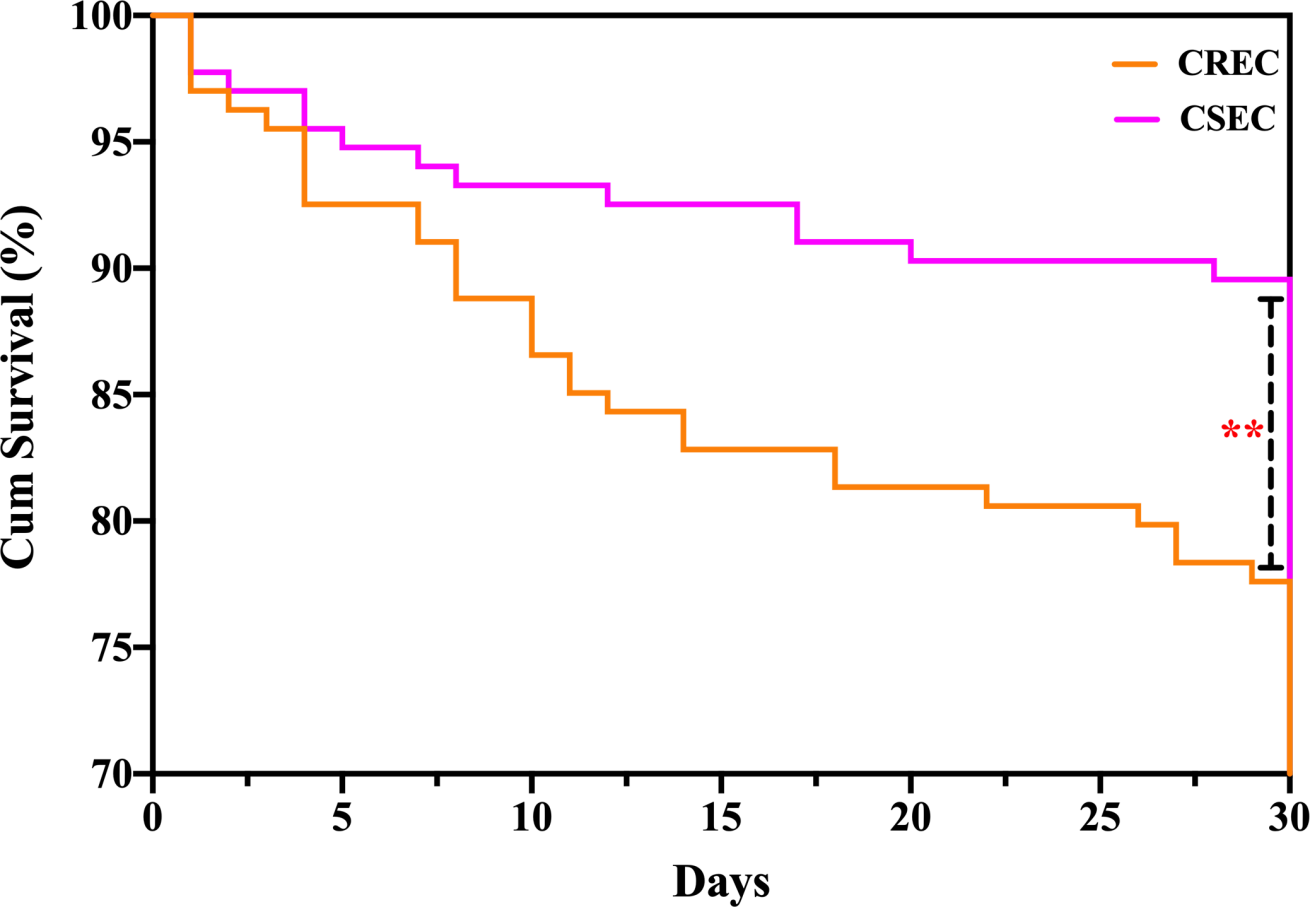
Kaplan‒Meier curves showing 30-day mortality in the CREC group versus the CSEC group (*P* = 0.009).

In our study, 30 patients (22.4%) died within 30 days of a positive CREC culture and 14 (10.4%) patients in the CSEC group died. The 30-day mortality rate was higher in the CREC group than in the CSEC group. There was a significant difference in the duration of hospitalization (*P* = 0.011). The clinical outcomes of the two groups are shown in Table 6. Variables associated with 30-day mortality in the Cox hazard model were CREC infections (HR = 2.40; 95% CI, 1.27–4.53; *P* = 0.007), carbapenem exposure (HR = 2.04; 95% CI, 1.01–4.13; *P* = 0.047), and gastric tube insertion (HR = 2.73; 95% CI, 1.34–5.54; *P* = 0.006). A Kaplan‒Meier survival analysis was performed, and the results are shown in Fig. 3.

**TABLE 6:** Clinical outcome comparison between the CREC group and the CSEC group^*a*^

Outcomes	CREC [n (%)]	CSEC [n (%)]	*P*
30-day mortality	30 (22.4)	14 (10.4)	0.008
In-hospital mortality	39 (29.1)	20 (14.9)	0.005
LOS (days), median (IQR)	29.5 (15,55)	19 (11.75,39.5)	0.011

^*a*^ CREC, carbapenem-resistant *Escherichia coli*; CSEC, carbapenem-sensitive *Escherichia coli*; LOS, length of hospital stays; IQR, interquartile range.

Thirty-nine patients (29.1%) in the CREC group died during hospitalization, whereas 20 patients (14.9%) in the CSEC group died during hospitalization. The in-hospital mortality rate was higher in the CREC group than in the CSEC group (*P* = 0.005). Univariate analyses comparing the clinical characteristics of patients with CREC infections who survived or died are shown in Table 7. The univariate analysis results indicated the variables associated with in-hospital mortality were as follows: age >65 years (OR = 1.52; 95% CI, 1.09–2.12; *P* = 0.02), digestive system diseases (OR = 1.57; 95% CI, 1.03–2.39; *P* = 0.04); sepsis (OR = 2.68; 95% CI, 1.24–5.79; *P* = 0.01); immuno-suppression (OR = 1.87; 95% CI, 1.20–2.93; *P* = 0.01); carbapenem exposure (OR = 1.53; 95% CI, 1.21–1.94; *P* = 0.002); central venous catheter insertion (OR = 2.07; 95% CI, 1.48–2.90; *P* ＜ 0.001); urinary catheter insertion (OR = 1.32; 95% CI, 1.08–1.61; *P* = 0.02); and ICU stay (*P* = 0.003). The multivariate conditional logistic regression analysis demonstrated that age >65 years (OR = 3.19; 95% CI, 1.29–7.90; *P* = 0.01), carbapenem exposure within 3 months prior to a positive culture (OR = 3.54; 95% CI, 1.26–9.98; *P* = 0.02), and central venous catheter insertion within 1 month prior to a positive culture (OR = 4.19; 95% CI, 1.70–10.31; *P* = 0.002) were independent risk factors for in-hospital mortality (Table 8).

**TABLE 7:** Univariate analyses of in-hospital mortality in the CREC group^*a*^

Variable	CREC survivors (*N* = 95) (n, %)	CREC non-survivors (*N* = 39) (n, %)	χ^2^/t/U	OR	95% CI	*P*
Demographic characteristics						
Sex, male	44 (46)	22 (56)	1.13	1.22	(0.86,1.73)	0.29
Age (years), (mean, SD)	57.3 ± 22.1	66.0 ± 18.5	2.18	—	—	0.03
Age > 65	40 (42)	25 (64)	5.36	1.52	(1.09，2.12)	0.02
Underlying disorder						
Respiratory diseases	48 (51)	26 (67)	2.91	1.48	(0.91,2.42)	0.09
Liver diseases	40 (42)	20 (51)	0.94	1.22	(0.83,1.79)	0.33
Urinary system diseases	42 (44)	24 (62)	3.32	1.39	(0.99,1.95)	0.07
Circulatory system diseases	58 (61)	30 (77)	3.09	1.26	(0.99,1.60)	0.08
Central nervous system diseases	39( 41)	21 (54)	1.83	1.31	(0.90,1.91)	0.18
Sepsis	10 (11)	11 (28)	6.54	2.68	(1.24,5.79)	0.01
Digestive system diseases	31 (33)	20 (51)	4.08	1.57	(1.03,2.39)	0.04
Diabetes mellitus	25 (26)	12 (31)	0.27	1.17	(0.66,2.09)	0.6
Malignancy	5 (5)	1 (3)	0.47	0.49	(0.06,4.04)	0.67
Antibiotic exposure within 3 months prior to CREC infection						
Third-generation cephalosporin	50 (53)	15 (38)	2.39	0.72	(0.47,1.12)	0.12
β-lactam inhibitor	23 (24)	14 (36)	1.89	1.48	(0.86,2.57)	0.17
Carbapenem	51 (54)	32 (82)	9.44	1.53	(1.21,1.94)	0.002
Quinolones	28 (29)	11 (28)	0.02	0.96	(0.53,1.73)	0.88
Tigecycline	7 (7)	7 (18)	3.31	2.44	(0.92,6.48)	0.12
Macrolides	5 (5)	0 (0)	2.13	—	—	0.14
Antifungal agents	5 (5)	4 (10)	1.1	1.95	(0.55,6.88)	0.29
Glucocorticoid	19 (20)	8 (21)	0.01	1.03	(0.49,2.14)	0.95
Surgical history and invasive procedures within 1 month prior to CREC infection						
Surgical history	50 (53)	24 (62)	0.89	1.17	(0.86,1.60)	0.35
Mechanical ventilation	34 (36)	20 (51)	2.76	1.43	(0.95,2.15)	0.10
Central venous catheter insertion	33 (35)	28 (72)	15.3	2.07	(1.47,2.90)	<0.001
Arterial catheters	30 (32)	14 (36)	0.23	1.14	(0.68,1.90)	0.63
Urinary catheter insertion	61 (64)	33 (85)	5.50	1.32	(1.08,1.61)	0.02
Drainage tube insertion	26 (27)	17 (44)	3.34	1.59	(0.98,2.59)	0.07
Gastric tube insertion	45 (47)	24 (62)	2.22	1.30	(0.94,1.80)	0.14
Related to hospitalization						
Prior hospital stays, median (IQR)	10 (3,98)	12 (3,173)	1616	—	—	0.25
ICU stays, median (IQR)	0 (0,75)	15 (0,131)	1269	—	—	0.003
LOS (days), median (IQR)	27 (3,148)	34 (3,193)	1760	—	—	0.65

^*a*^ SD, standard deviation; CREC, carbapenem-resistant *Escherichia coli*; ICU, intensive care unit; LOS, length of hospital stays; IQR, interquartile range; —, no result.

**TABLE 8:** Multivariate analyses of in-hospital mortality in the CREC group^*a*^

Variable	β	SE	Wald (χ^2^)	OR	95% CI	*P*
Age >65	1.16	0.46	6.27	3.19	1.29–7.90	0.02
Carbapenem	1.26	0.53	5.72	3.79	1.17–12.31	0.02
Central venous catheter insertion	1.43	0.46	9.71	4.19	1.70–10.31	0.002

^*a*^ CREC, carbapenem-resistant *Escherichia coli*.

## DISCUSSION

To the best of our knowledge, few studies have evaluated the risk factors for the acquisition of CREC infection, with most reports focusing on the mechanisms of carbapenem resistance in CRE (25–28). Therefore, the aim of this matched case–control study was to assess the potential risk factors for CREC infections.

In this study, we found that CREC infections were mainly concentrated in the ICU, followed by the emergency department in the general medical ward and the neurosurgical department in the surgical ward. Patients in the ICU have serious underlying diseases and require life-support systems and broad-spectrum antibiotics (29). Patients with various infectious diseases are often treated in the emergency department. Patients undergoing craniocerebral surgery need to have a drainage tube in place for a long time and use broad-spectrum antibiotics because of potential intracranial infections.

All the CREC strains were resistant to ampicillin, cefazolin, cefuroxime, ceftriaxone, and ciprofloxacin. Antibiotic resistance was severer in the CREC group than in the CSEC group, and the differences between the antibiotics listed in Table 2 in the two groups were statistically significant. However, these strains were relatively susceptible to amikacin. Considering the aforementioned results and the individual clinical conditions, the treatment of CREC infections was optimal.

Our results indicate that exposure to antibiotics (third-generation cephalosporin and carbapenems), glucocorticoids, and surgical history are risk factors for CREC infection.

Studies have reported a close association between CREC infection and antibiotic use, particularly carbapenem exposure (30–33). Our study showed that prior carbapenem use was an independent risk factor for CREC infection, which is consistent with the results of most previous studies. Acquisition of the ability to produce carbapenemase is the main cause for carbapenem resistance, which can easily result in nosocomial spread (34). Tian et al. demonstrated that the most prevalent carbapenemase gene in CREC in China is blaNDM, followed by blaKPC-2 (10). However, Dagher et al. found that the predominant carbapenemase detected was OXA-48 in Lebanon (35), and that carbapenem exposure may induce the emergence of these resistance-conferring genes. In our study, third-generation cephalosporin exposure within 3 months was also a risk factor for CREC infection, indicating that CREC infection can be induced not only by the use of carbapenem but also by the use of other drugs. Thus, we need to strengthen the management of antibiotics for inpatients, and treatment with high doses for controlled durations is a better way to limit the risk of infection (32).

Unlike other studies, our study revealed that previous use of glucocorticoids was also a risk factor for CREC infection, possibly because patients with CREC infections have serious underlying diseases and are treated with glucocorticoids. Second, the use of glucocorticoids destroys the intestinal microenvironment, kills sensitive strains, and promotes the overgrowth of drug-resistant strains, which promotes the shift of opportunistic pathogens to pathogenic bacteria.

It is not surprising that surgical history was a risk factor for CREC infection, which is consistent with previous studies emphasizing the importance of aseptic operations in patient care (36). The asepsis technique is an important strategy for preventing CREC infections.

In our study, gastric tube insertion was identified as an independent risk factor for 30-day mortality. Meta-analyses revealed that the use of medical devices, including gastric tubes, presents the highest aggregated estimate (37). Furthermore, multiple studies have proposed that CREC colonization of the gastrointestinal tract contributes to subsequent CREC infection in afflicted individuals (38). Additionally, a significant proportion of patients undergoing gastric tube insertion exhibited gastrointestinal dysfunction and a compromised nutritional status, which could facilitate bacterial translocation in the gastrointestinal tract and potentially fostering pathogenic bacterial infections, including CREC. CREC infection and carbapenem exposure were independent risk factors for 30-day mortality. Therefore, the judicious use of carbapenems in antibiotic stewardship programs, reduction of invasive procedures, and prevention of CREC infections are effective ways to reduce 30-day mortality.

Patients with CREC infections who died were significantly older than surviving patients with CSEC infection. Possible reasons for this include more frequent healthcare exposure and antibiotic exposure among older adults (39). Central venous catheter insertion was an independent risk factor for in-hospital mortality in patients with nosocomial CREC infections. The most likely reason for this is that invasive procedures can damage the mucosa and increase the incidence of CREC infection because the majority of the bacteria are able to pass through the mucosal barrier to other sites (40). Carbapenem exposure was also a risk factor for in-hospital mortality in patients with CREC infection. The possible reasons for this are the same as mentioned previously.

Recently, an increasing number of studies have focused on active screening for CRE, enabling early detection, isolation, and intervention in high-risk departments or patients. The World Health Organization (WHO) and the US Centers for Disease Control (CDC) recommend screening for CRE based on local epidemiological conditions (41, 42). In our hospital, nosocomial CREC infections tend to occur in specific departments and in patients with risk factors, suggesting that those higher-risk departments should actively screen for CREC.

This study has several limitations that should be considered. First, a molecular epidemiological investigation of CREC was not performed; thus, we could not assess whether there were any outbreaks during the study period and whether different drug resistance mechanisms resulted in different clinical outcomes; future studies should include molecular epidemiological investigations. In addition, analyzing risk factors through case–control studies has the potential to increase the utilization of antibiotics, which can introduce statistical selection bias. This is due to the fact that certain antibiotics may inhibit or eliminate certain strains of CSEC, while having no effect on CREC. Consequently, the frequency of antibiotic usage in CSEC strains may decrease, indirectly amplifying the risk factor for CREC (43). Therefore, attention should be paid to the selection of the control group, as choosing an antibiotic-sensitive group as the control group may increase the risk of antibiotic exposure (44). Notably, this was a single-center study with a modest sample size. Hence, it is imperative to conduct prospective, multicenter, large-sample clinical trials to validate our findings.

### Conclusion

The aim of this study of nosocomial CREC infection was to establish a foundation for the formulation of efficacious control strategies. In our hospital, CREC infections exhibited a focal distribution across specific departments, such as the ICU, emergency department, and neurosurgery department, underscoring the need to implement interventions such as hand hygiene protocols and environmental disinfection measures to limit CREC transmission. Paying close attention to patients who have used carbapenem and cephalosporins for a long time, the rational use of antibiotics, and the reduction of invasive procedures are effective measures to reduce CREC infection and 30-day or in-hospital mortality. These findings provide valuable evidence for the development of anti-infective therapy, infection prevention, and control of CREC-positive infections.

## Data Availability

The dataset used and/or analyzed during the study is available from the corresponding author upon reasonable request.
